# Supplementary material for: Association between oral microbiome and seven types of cancers in East Asian population: a two-sample Mendelian randomization analysis
Source: Front Mol Biosci. 2023 Nov 21;10:1327893. doi: 10.3389/fmolb.2023.1327893 (PMC10702768; doi:10.3389/fmolb.2023.1327893)
Supplement: Supplementary file 1 [file DataSheet1.ZIP › Supplementary Materials/MR plots of saliva/saliva═╝/Breast cancer/pheno.347_to_breast cancer_forest.pdf]

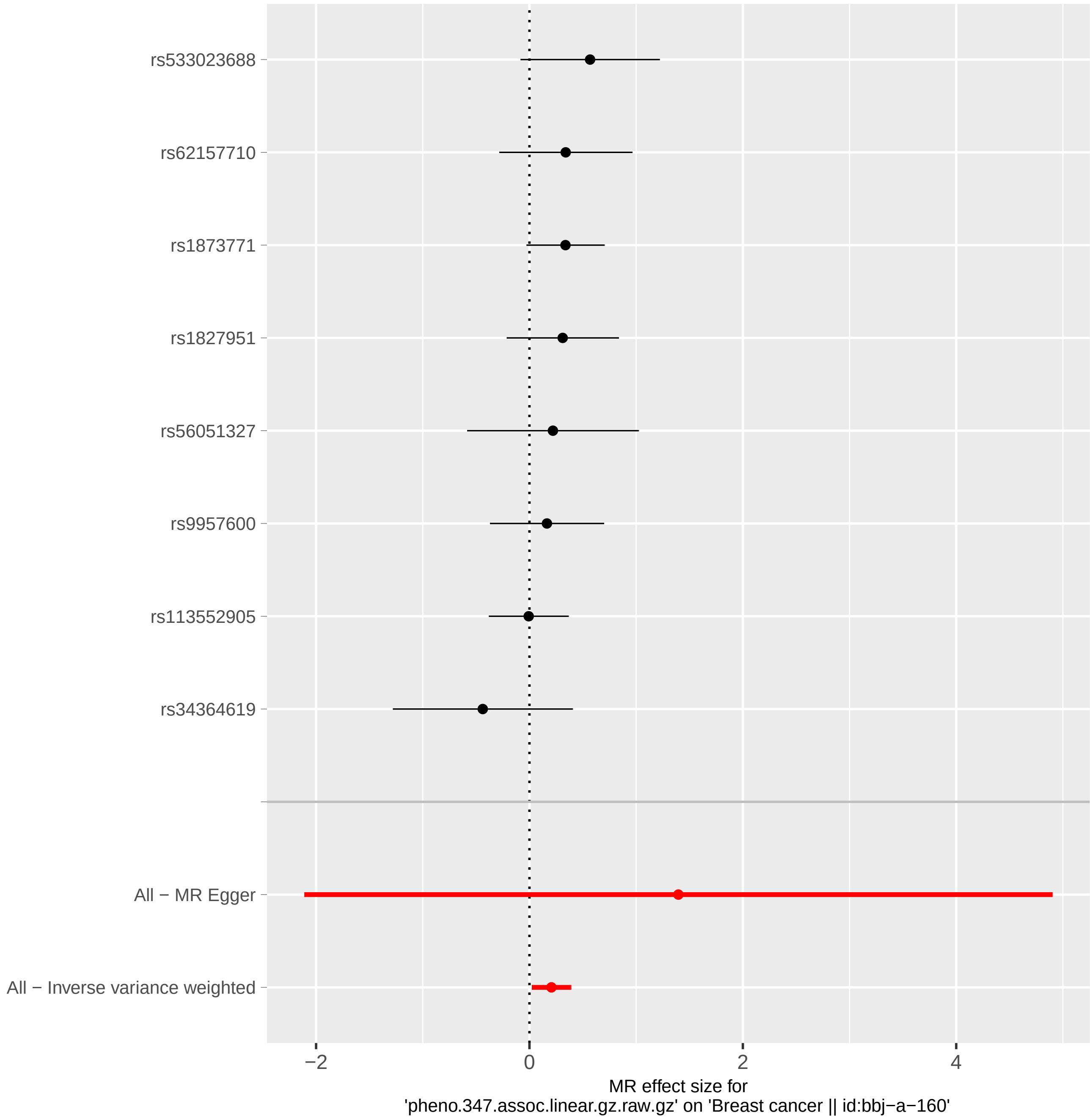
rs533023688
rs62157710
rs1873771
rs1827951
rs56051327
rs9957600
rs113552905
rs34364619
All − MR Egger
All − Inverse variance weighted
−2
0
2
4
MR effect size for
'pheno.347.assoc.linear.gz.raw.gz' on 'Breast cancer || id:bbj−a−160'
